# Letting patients lead? Practitioners’ experiences of using DIALOG+ in psychosis outpatient care – patient involvement, collaborative dynamics, and professional roles

**DOI:** 10.1186/s12888-026-08633-6

**Published:** 2026-09-17

**Authors:** Marcus Lundmark, Matilda Cederberg, Ann-Christine Andersson, Andreas Gremyr

**Affiliations:** 1 Department of Psychotic Disorders, Sahlgrenska University Hospital, Gothenburg, Sweden; 2 The Jönköping Academy for Improvement of Health and Welfare, Jönköping University, Jönköping, Sweden; 3 Institute of Health and Care Sciences, University of Gothenburg, Gothenburg, Sweden; 4 Region Jönköping County, Jönköping, Sweden

**Keywords:** Co-production, DIALOG+, Person-centred care, Patient participation, Professional-patient relations, Psychotic disorders, Qualitative research, Schizophrenia

## Abstract

**Background:**

Severe mental illness, such as psychosis, significantly affects many areas of life. Involving patients in their care may enhance engagement and support recovery by ensuring that treatment focuses on their expressed needs. DIALOG+ is an intervention, supported by a digital tool, aimed at facilitating patient involvement in treatment by incorporating self-assessment and a problem-solving approach during routine clinical encounters and promoting a collaborative and patient-centred approach to care. The aim of this study was to explore mental health practitioners’ (MHPs) experiences of how DIALOG+ influenced patient involvement and the dynamics of collaboration in outpatient psychosis care.

**Methods:**

A qualitative study was designed to describe MHPs’ experiences of using DIALOG+ in psychosis outpatient care in Sweden. Fourteen MHPs who had used DIALOG+ three times or more were included in the study. Four focus groups were conducted using semi-structured interviews and analysed through reflexive thematic analysis.

**Results:**

The analysis of MHPs’ experiences resulted in three themes: “The patient’s voice and engagement,” “The relationship between patient and mental health practitioner,” and “Professional challenges and changes in the practitioner’s role.” DIALOG+’s structured approach made it easier for patients to express their needs and take a more active role in the conversation, sometimes leading to increased patient engagement. The intervention also seemed to balance the conversation by sharing responsibility for decisions and follow-up between patients and practitioners. At the same time, practitioners reflected on challenges when letting the patient lead the conversation, which could result in time constraints and the need to balance patient-led agendas with professional responsibility.

**Conclusion:**

DIALOG+ has the potential to support the operationalisation of co-production in outpatient psychosis care by increasing patients’ influence over the agenda in routine clinical encounters. It provides a structured framework for shared reflection and action planning, while also making visible potential differences between patients’ preferences and practitioners’ clinical assessments that can be negotiated. However, its impact depends on how the approach is enacted in practice. Supporting practitioners in navigating shifts in professional roles and authority may therefore be important for realising the full potential of the intervention.

**Supplementary Information:**

The online version contains supplementary material available at https://doi.org/10.1186/s12888-026-08633-6.

## Background

Participation in treatment decisions for patients with mental illness has been associated with improved empowerment, treatment adherence, self-efficacy, and recovery-related outcomes [12, 25]. Despite policy-level endorsement of person-centred approaches and shared decision-making, implementation in routine psychiatric care remains inconsistent. A recent national evaluation in Sweden identified a substantial gap between formal routines promoting patient participation and their actual use in clinical practice (The National Board of Health and Welfare, [22]).

These challenges are evident in psychosis care, where people with psychotic disorders report limited involvement in decisions regarding their care [10, 11]. Psychotic disorders, including schizophrenia, affect approximately 0.75% of the population [18] and are associated with cognitive impairments, functional limitations, increased somatic comorbidity, and reduced life expectancy ([7, 24]; World Health Organization, n.d.). Concerns regarding risk, fluctuating insight, and decisional capacity may reinforce traditional professional authority and complicate efforts to involve patients in clinical encounters. Qualitative research has shown that clinicians may struggle to balance patient autonomy with professional responsibility, particularly when patient preferences appear to conflict with clinical judgement [9, 13]. Shared decision-making in psychosis has therefore been described as positioned “midway on a continuum between paternalistic decision-making practices by clinicians and autonomous, informed decision-making by patients” ([23], p. 23).

Increasing patient participation may require changes not only in patients’ involvement but also in how professionals enact their roles. In mental health care, concerns about power, patients’ best interests, and professional responsibility have been identified as key barriers to shared decision-making [1]. More broadly, shifts towards less hierarchical relationships and shared responsibility may challenge established professional identities and require professionals to negotiate new relational roles [6].

Co-production has emerged as a conceptual framework seeking to move beyond this dichotomy. It emphasises collaboration between patients and professionals in designing, delivering, and evaluating care [2, 3]. At the individual level, co-production entails the integration of patient-defined outcomes, individual goal-setting, and joint responsibility for action [8]. However, putting these principles into everyday clinical practice requires more than theoretical endorsement. It requires practical tools that help shift influence within the clinical encounter. Without such tools, person-centred care may remain an ideal rather than something that is actually carried out in practice.

One approach intended to support this shift is DIALOG+, a structured, computer-assisted intervention developed at Queen Mary University of London in collaboration with East London NHS Foundation Trust [20]. By incorporating patient-defined priorities and formalising joint reflection and decision-making, DIALOG+ may provide a concrete structure for operationalising co-production on the individual level in routine care. In this study, co-production is understood as the collaborative process through which patients and professionals jointly assess priorities, make decisions, and develop care within the clinical encounter [2, 8]. DIALOG+ integrates patient self-assessment of life domains and experience of care with a solution-focused dialogue and joint action planning during routine meetings. In each session, patients rate their satisfaction across several life and treatment domains, identify areas they wish to prioritise, and engage in a structured problem-solving process leading to agreed actions. The intervention embeds review of previous ratings and actions over time, thereby creating continuity [21]. Rather than functioning solely as a structured assessment or goal-setting tool, DIALOG+ actively involves patients in identifying priorities, selecting areas for discussion, and jointly developing actions to address issues they consider most important. Previous research has demonstrated the effectiveness of DIALOG+ in improving quality of life and other treatment outcomes [20]. In a mixed-methods process evaluation of DIALOG+, Omer et al., [19] found that patients experienced increased self-reflection and empowerment, while some clinicians reported challenges in adhering to the structured format. Omer et al., also found that clinicians perceived DIALOG+ as facilitating more focused discussions and greater patient involvement in identifying problems and solutions, while patients assumed responsibility for most agreed actions. However, the primary aim of that study was to explore the mechanisms through which DIALOG+ improved patient outcomes rather than how practitioners experienced changes in their professional role or collaborative practice. In Sweden, DIALOG+ has been translated and adapted for psychiatric practice [15], and implementation in outpatient psychosis services has generated increasing experience with the intervention. A recent qualitative study exploring patients’ experiences in psychosis outpatient care found that the intervention supported articulation of needs and strengthened involvement in treatment [16]. Although previous research has examined patient outcomes and explored both patient and clinician experiences of DIALOG+, the implications of these interventions for practitioners’ professional roles have received less attention. In particular, little is known about how practitioners experience and negotiate changes in agenda-setting, authority, and responsibility during routine psychosis care in the Swedish context. Understanding these dynamics is important, as operationalising co-production on the individual level depends not only on patient activation but also on how professional influence is negotiated and enacted within routine clinical encounters.

Therefore, the aim of this study was to explore MHPs’ experiences of how DIALOG+ influenced patient involvement and the dynamics of collaboration in outpatient psychosis care.

## Methods

### Study design and setting

This study used a qualitative design, involving focus group interviews with MHPs working in outpatient psychosis care. The study followed the Consolidated Criteria for Reporting Qualitative Research (COREQ) checklist to ensure transparency and rigour in reporting, see supplementary file 1.

The study was conducted within the Department of Psychotic Disorders at Sahlgrenska University Hospital in Gothenburg, Sweden. The department comprises seven specialist psychiatric outpatient clinics providing care for individuals with psychotic disorders. Together, these clinics are responsible for approximately 3,000 patients and conduct around 30,000 clinical contacts per year. The clinics provide long-term, specialist-level psychiatric care based on a case management model with Flexible Assertive Community Treatment elements [26]. Care is delivered by interdisciplinary teams consisting of psychiatrists, nurses, social workers, occupational therapists, psychologists, and either physiotherapists or health coaches. The treatment focuses on symptom management and includes both pharmacological and psychosocial interventions. The care process is structured around regular follow-ups and individual care planning with the goal of promoting stability and recovery.

### The DIALOG+ intervention

DIALOG+ is an evidence-based, computer-assisted intervention designed to enhance the therapeutic value of routine clinical encounters [20]. A DIALOG+ session is supported by a digital application accessed on a computer and used during patient visits, either in person or during virtual meetings.

During such a DIALOG+ session, patients are asked to rate their satisfaction on a scale from 1 (lowest) to 7 (highest) across eight areas of life: mental health, physical health, employment situation, accommodation, leisure activities, relationships with partner/family, friendships, and personal safety. Patients also provide satisfaction ratings for three aspects of care: medication, practical support, and meetings with professionals. In addition, patients indicate areas in which they would like additional support.

**Fig. 1:**
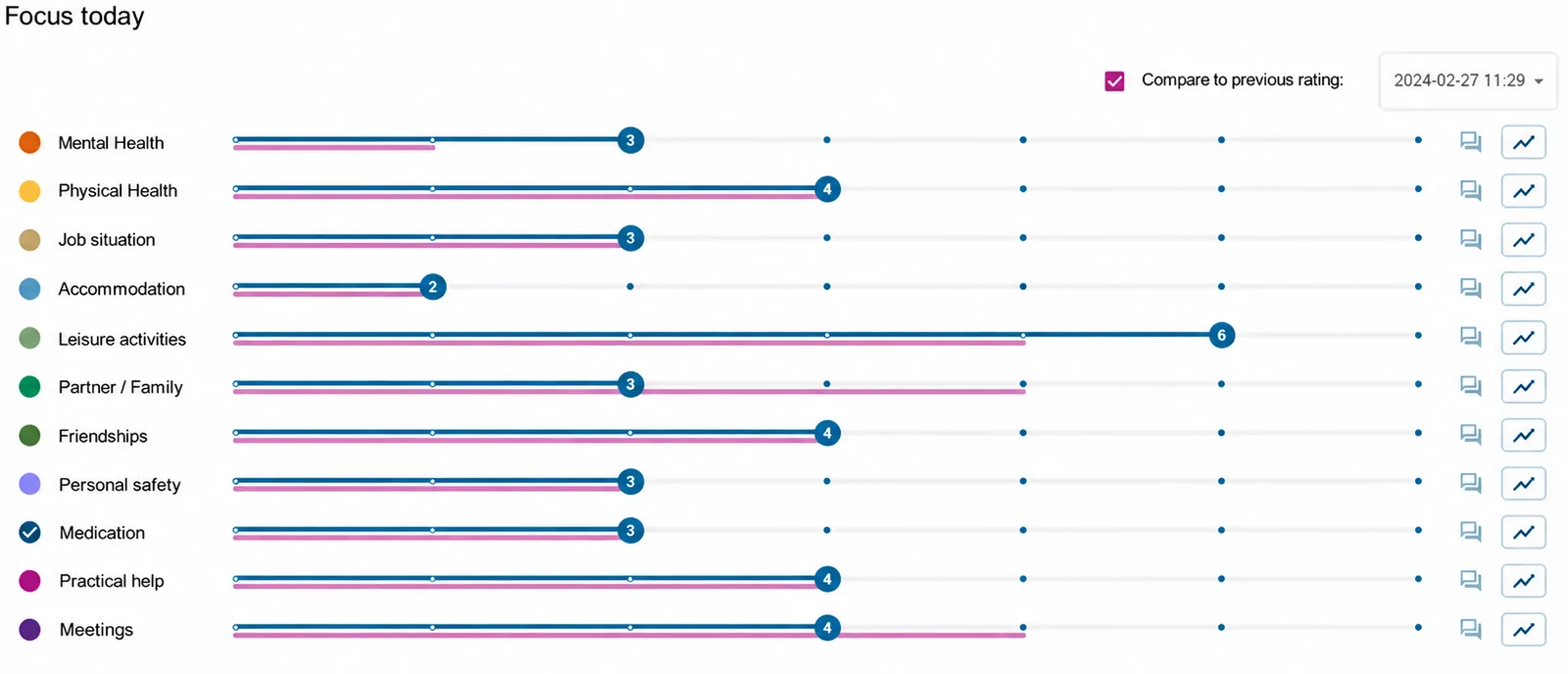
Illustrative example of the DIALOG+ interface showing patient satisfaction ratings, with the current session (top blue line) compared with a previous session (lower pink line). The conversation areas are listed on the left

The MHP and patient review an overview of the ratings, discuss the results, and jointly decide which areas to focus on during the meeting. If DIALOG+ has been used in previous sessions, current ratings can be compared with earlier ones, offering a longitudinal perspective on the patient’s progress (see Figure 1). For each area in focus, a four-step solution-focused approach is applied: (1) Understanding: Clarify the patient’s current concerns, (2) Looking forward: Identify scenarios for improvement, (3) Exploring: Discuss potential actions to address concerns, and (4) Agreeing: Decide on concrete actions to improve the patient’s condition and social situation.

At the end of the session, a summary of the ratings and agreed actions is shown and can be printed for the patient to bring home. A more detailed account of the DIALOG+ process, including screenshots of the interface, see supplementary file 2.

### Recruitment and participants

DIALOG+ was delivered by the MHPs responsible for patients’ ongoing care within the case management model, while psychiatrists were consulted when issues requiring medical assessment or expertise, such as medication management, were identified. MHPs were eligible to participate if they had conducted at least three DIALOG+ sessions. Thirty-one individuals from seven outpatient units met the inclusion criteria, of whom 14 agreed to participate in the study. Recruitment was carried out across the seven outpatient clinics within the department, and MHPs from five of these were ultimately represented in the focus group interviews. The participants represented different professional roles, including nurses (*n* = 7), mental health support workers (*n* = 5), and social workers (*n* = 2). Nine participants were women, and five were men. Their experience working in outpatient psychosis care ranged from 1 to 44 years, with a mean of 13.5 years. The MHPs had used DIALOG+ between 3 and 23 times, with a median of 6 sessions. Participation was voluntary, and all participants provided informed verbal consent. This information was communicated during the recruitment process and reiterated at the beginning of each focus group interview.

### Data collection

Four focus group interviews were conducted, with one conducted virtually for practical reasons. Three groups included four participants each, and one group included two. The interviews took place during September and October 2023 and lasted between 60 and 100 min, averaging 77 min. The duration reflected the relatively small size of the focus groups. All topics were explored before the interviews were concluded. To minimise potential bias or influence, two authors (ACA and MC), who had no prior relationship to the participants, moderated the interviews. ML did not participate in the focus group interviews.

A semi-structured interview guide was used, which was initially developed by ML and iteratively refined in collaboration with ACA, MC, and AG (see Supplementary file 3). The interview guide was informed by findings from earlier interviews with patients [16] and was designed to elicit a broad range of perspectives on the use of DIALOG+, including perceived benefits and challenges for both MHPs and patients. The interview guide served as a flexible framework. Although predefined topics were covered in each focus group, participants were encouraged to discuss issues freely, elaborate on each other’s reflections, and introduce new topics as they emerged. Follow-up questions were used to facilitate deeper exploration rather than to constrain discussion. All interviews were audio-recorded and transcribed verbatim in Swedish. Selected quotations were translated into English for reporting purposes.

### Data analysis

The interviews were analysed using reflexive thematic analysis (RTA) as described by Braun and Clarke [4]. This method was chosen to enable researcher reflexivity and interpretation, allowing for a deeper analysis of patterns and underlying meanings that may not be immediately evident in the surface-level content of the discussions. The focus group interviews were conducted at a relatively early stage of the implementation process at the clinic. At that time, 60 practitioners and 180 patients had used DIALOG+ at least once, and approximately 300 DIALOG+ sessions had been carried out in total. ML and MC jointly carried out the initial analysis, beginning with a thorough reading of all transcripts to familiarise themselves with the data. NVivo 14 was used to support the analysis process. Initially, an inductive approach was taken, with open coding to capture the MHPs’ experiences of using DIALOG+. These initial codes were then organised into a range of categories. As the analysis progressed, some categories emerged as particularly prominent across all focus groups, especially those relating to patient involvement. In discussions with ACA, it was decided to narrow the analysis to focus on experiences of how the use of DIALOG+ affected patient involvement and what clinical expressions this could take. A second round of coding was undertaken, focusing particularly on these aspects. To provide a balanced account, the analysis included both experiences describing how DIALOG+ supported patient involvement and experiences highlighting challenges associated with patient involvement in routine clinical encounters.

Themes and sub-themes were developed through an iterative dialogue between ML and MC, with feedback and input from ACA throughout the process. ACA contributed to theme formulation and the reallocation of data extracts. AG also provided input during the later stages of analysis. As the study employed reflexive thematic analysis, the analytical focus was on the content and meaning of participants’ accounts rather than on interactional processes within the focus groups, such as consensus or disagreement.

The analysis was conducted across the dataset as a whole and did not seek to compare perspectives between professional groups or according to participants’ level of experience with DIALOG+, as all participants shared the same function in routinely delivering DIALOG+ within the case management model and the intention was to identify shared patterns of meaning across the dataset. The analysis was reflexive, with the researchers actively engaging with the data and their own interpretations. ML and MC held frequent discussions to challenge and refine their interpretations, ensuring that the findings reflected the data rather than personal assumptions.

Given the reflexive nature of thematic analysis, it is important to acknowledge the researchers’ backgrounds, as their disciplinary perspectives and prior experiences may influence the interpretation of the data. In particular, ML’s familiarity with several of the participants required careful consideration to ensure that existing relationships did not shape the analytic process. This was addressed through ongoing reflexivity and a conscious effort to interpret the data based on what was expressed in the interviews, rather than prior knowledge of the individuals. ML is a psychiatric nurse with a master’s degree in quality improvement and has extensive experience in psychosis care. ML led the implementation of DIALOG+ within the participating outpatient services and was involved in training staff in the intervention. MC is a psychologist with clinical experience in psychosis care and a PhD in health care sciences, whose research focused on person-centred care. AG, a psychologist, conducted his doctoral research in Learning Health Systems and has contributed to the Swedish adaptation of DIALOG+ since its early stages. ACA is a senior researcher with expertise in qualitative research and quality improvement. ACA and MC were not involved in the implementation or delivery of the intervention.

## Results

The analysis generated three overarching themes, each comprising two interrelated sub-themes. The three themes were: (1) “The patient’s voice and engagement”, (2) “The relationship between patient and mental health practitioner”, and (3) “Professional challenges and changes in the practitioner’s role”, as shown in Table 1.

**Table 1:** Overview of themes and sub-themes identified in the reflexive thematic analysis

Theme	Sub-theme 1	Sub-theme 2
1. The patient’s voice and engagement	Support self-expression and identification of needs	Strengthened confidence and motivation
2. The relationship between patient and mental health practitioner	Power relations and relational dynamics	Shared responsibility
3. Professional challenges and changes in the practitioner’s role	Challenging situations	Benefits of structured conversations

### Theme 1: The patient’s voice and engagement

This theme focuses on how MHPs experienced DIALOG + as influencing patients’ ability to identify and articulate their own needs, as well as supporting participation and self-expression during clinical conversations. It also encompasses practitioners’ reflections on how the intervention may have contributed to greater patient involvement.

#### Support self-expression and identification of needs

Practitioners described how the use of DIALOG+ supported patients in expressing their individual needs and priorities during routine encounters. The structured yet open format opened up new kinds of discussions, allowing patients’ experiences and concerns to become more central in the conversation. Practitioners described this as a shift from pre-defined clinical agendas to patient-led agendas, focusing on what matters most to patients. The accounts indicate how the intervention influenced the topics discussed during sessions, as patients’ own perspectives contributed to shaping the content of care. Practitioners highlighted that this patient-focused approach sometimes captured needs that might otherwise have remained unspoken, broadening the scope of what was addressed in care:

In our first session, something came up that we’d never heard from the patient before—he needed help applying for financial support because of holes in the wall at home. Suddenly, we involved the social worker, applied for support, and he actually benefited from it. Without the structured approach, we probably would never have known. It was a win-win

This shift was also evident in how patients responded to the process of selecting priorities:

What’s positive is what they say—‘Now I get to choose. Now I get to focus on what matters to me.’ We’re not sitting with all the keywords from the care plan, trying to define goals and figure out what we’re supposed to capture. This actually becomes the foundation, based on what the patient has selected.

A key aspect that further seemed to enhance patients’ sense of ownership was the opportunity to write their own input in their own words. Practitioners described how even subtle editorial choices on their part could influence meaning, and how handing over control could empower patients:

If the patient isn’t writing and says they want help with housing, for example, and then you’re the one who writes it down… you end up rephrasing it to keep it short. ‘Is it OK if I write it like this?’ you ask, and then you’ve already put a value on what they meant. But if they write it themselves—even if it’s a long sentence or just one word—it’s in their own words. I haven’t validated or altered it at all. That’s better, I think. I’d even recommend it.

#### Strengthened confidence and motivation

Practitioners described how the use of DIALOG+ contributed to a broader sense of ownership and motivation among patients. Some practitioners expressed that the visualisation of patient ratings, especially when used over time, seemed to help patients recognise progress, which in turn led to engagement and motivation for change. It also appeared to strengthen patients’ confidence in their ability to influence their situation and their overall sense of control. Over time, some practitioners described how patients’ motivation and engagement gradually increased as they became more familiar with the method. For some, this was described as a reminder of patients’ strengths, and at times even as a somewhat unexpected outcome.

It becomes very concrete. They like being able to follow what’s happening, and it becomes really visible. It gives them help and also gives them perspective. And maybe even a bit of increased self-esteem from feeling more in control of their life.

Graphical representations and repeated use of the tool were seen as important elements for increasing engagement:

That’s why the layout with graphs and everything is good, I think. It makes it clear, and for many patients, it becomes even clearer that something is happening. Because that’s not always obvious when you wake up and feel like it’s the same today as it was yesterday. It’s motivating too, actually.

Giving patients clear control over the agenda was also seen as a source of motivation and a ‘selling point’ for the intervention:

In some of the cases where I’ve done the ratings, the fact that the patient is in control is what has made it work. When you explain that this isn’t something we’re directing—if you want to do this, we’ll do it—it’s your choice what we talk about. That’s actually been a selling point. Especially in that first session—that’s what got him on board.

### Theme 2: The relationship between patient and mental health practitioner

This theme captures practitioners’ reflections on how the use of DIALOG+ appeared to influence the relational dynamics with their patients. The sub-themes “Power relations and relational dynamics” and “Shared responsibility” reflect both the perceived redistribution of influence and the ongoing negotiation of professional responsibilities.

#### Power relations and relational dynamics

When allowing patients to address their needs and choose topics for the conversation, it appeared to affect the traditional roles and responsibilities, since practitioners had to hand over some control over the conversation, leading to a greater sense of uncertainty compared with a traditional meeting. They also described various ways within the DIALOG+ format for involving patients during sessions, which appeared to alter the power relations, from allowing patients to present their ratings and needs projected on the wall, to letting them use the keyboard and mouse themselves instead of the practitioner. For example, one practitioner described allowing the patient to handle the laptop:

I usually let the person use my laptop on their lap, and then he controls it. He writes himself and fills in the scales, so it becomes more his document than if I sit there writing. I project it onto the screen so we can see it together, but it’s the patient who does all the work.

This not only appeared to alter who handled the technology, but also helped the practitioners to hold back their impulse to steer the conversation. Some practitioners reflected that this led to a more collaborative and equal dynamic, but also recognised a tension between their professional responsibility to bring up issues they considered important and the patients’ own priorities and expressed needs.

When patients were more engaged in choosing topics, practitioners found themselves taking a step back and handing over some control to the patient:

It’s very much about the patient saying themselves what areas are important… otherwise, I might have directed things in a certain way or thought, ‘Well, this is what we’ll follow up now that we’re sitting here.’ But maybe they want to talk about something we otherwise would have missed. I might think something is very important and probably would have focused on that, but the patient wants to talk about something completely different, and then I have to be more flexible than I would be in a standard conversation.

One practitioner described that the power held by the practitioner in relation to the patient is inherent and how the use of DIALOG+ might help reduce that:

Of course, we sit in a position of power; there’s no getting around that. But DIALOG+ invites a conversation around different themes where we don’t judge; we just gather information to help us stay focused. In my experience, the sessions become more focused on what the patient chooses to talk about after you’ve used the system a few times.

Beyond routine settings, some practitioners saw potential for DIALOG+ to improve therapeutic alliances even in contexts marked by coercion and limited patient agency:

DIALOG+ might be useful for building alliances. It doesn’t necessarily have to lead to necessary actions. Perhaps what’s necessary is that we achieve a better alliance despite all the compulsion. It can go in that direction, too.

#### Shared responsibility

In contrast to broader shifts in power and relational dynamics, this sub-theme focuses more specifically on practitioners’ experiences of sharing and coordinating responsibility in clinical practice. Participants described that the use of DIALOG+ seemed to create a sense of shared responsibility in the conversation, where patients and practitioners reflected together, distributed tasks, and made joint decisions. They discussed moving away from the traditional role of the MHP as the sole problem-solver, towards a more collaborative approach where MHPs and patients solve problems together.

When patients were encouraged to think through issues themselves, practitioners appeared to observe a deeper sense of engagement and ownership:

I tell him: think about how you want things to be, what you want help with, how you see the situation, and how you plan to solve it. And he usually arrives at an idea quite quickly. It becomes more meaningful because I haven’t interfered—I’ve listened, and we’ve exchanged ideas, but the decision is his. The whole structure really helps in making it person-centred.

Practitioners also reflected on the practical distribution of tasks and responsibilities as a way of activating patients’ resources and networks. One participant described how this could be an opportunity to identify who should take the lead on different aspects of care:

What I like—this came to mind with one of my patients—is asking: ‘What can you do and what should I do?’ And it’s quite beautiful because it mobilises the patient’s own strength. Like, ‘Can your mum help?’ ‘Well, I can still make the call myself.’ So it’s about dividing up who does what.

Rather than solving problems for the patient, practitioners highlighted the importance of stepping back to allow space for the patient’s own thought process. This was often described as both challenging and essential:

As I’ve said many times, let the patient reflect on it themselves. I think it’s incredibly important that they take the time to think. That we don’t do that for them. I find it easy to jump in and become the problem-solver all the time. So… no, there’s no rush.

This approach appeared to nurture patients’ sense of self-determination and foster growth over time:

Getting patients to talk and make their own choices—that’s self-determination, and that’s what we strive for. I think they grow each time, again and again.

### Theme 3: Professional challenges and changes in the practitioner’s role

This theme describes practitioners’ experiences of how the structured format of DIALOG+ influenced their professional roles. The first sub-theme, ‘Challenging Situations’, focuses on the difficulties practitioners faced when using the intervention, such as time constraints, patient ambivalence, and tensions between structure and flexibility, which at times seemed to limit opportunities for participation. In contrast, the second sub-theme, ‘Benefits of Structured Conversations’, highlights how the structured format within DIALOG+ was perceived to support patient involvement by offering clarity, consistency, and focus on patients’ needs.

#### Challenging situations

Practitioners experienced various challenges when using DIALOG+ in their clinical work, such as time limitations, patient ambivalence, and cognitive difficulties, which sometimes made it difficult to complete a session as intended. Practitioners further reflected on the need to balance between a person-centred approach and the structured format of a DIALOG+ session. While the built-in structure of the intervention was valued by most participants, it also seemed to require clinical sensitivity, critical reflection on practitioners’ own roles, and the ability to adjust to each patient’s current situation. This highlights the complexity that may arise when using these kinds of structured interventions in clinical practice.

One recurring issue concerned the amount of time required to carry out a full session, especially with patients who had cognitive impairments or who engaged in lengthy reflections on each topic. As one practitioner explained:

Many of those I’ve done this with often get stuck discussing each part, so by the time we’re supposed to choose a focus area, there are only ten or perhaps five minutes left. Sessions could, therefore, feel rushed or incomplete, especially in busy clinical settings.

Time constraints were described not only as logistical challenges but also as shaping the quality of the conversation. In situations where sessions had to be expedited, some practitioners felt their own interpretations risked dominating the conversation:

If the patient wants to describe their housing situation, I’ll change the wording just to make it faster. Then it becomes my session, not theirs.

Another issue raised in practitioners’ accounts concerned situations where a patient’s self-assessment appeared to diverge from the practitioner’s understanding of their situation. This sometimes prompted reflection on how professional authority might shape, or potentially constrain, the patient’s autonomy.

Even though they’re not satisfied, they rate themselves high sometimes… and I know we’re at a “2” if we’re honest. But should I say that out loud? Then I’m the one steering.

In some cases, the structured nature of DIALOG+ was described as restrictive rather than helpful. Practitioners noted that certain patients did not resonate with the intervention, either because they could not relate to the predefined life areas or because they had other pressing concerns. As one participant explained:

They feel forced into discussing certain areas they don’t find relevant. It’s like they get boxed into a corner and you have to gently keep them within the focus areas.

Some practitioners described adapting the use of DIALOG+ to suit the individual patient’s needs, only focusing on selected areas, or allowing more unstructured dialogue. These adaptations were framed as a way to preserve person-centredness while still benefiting from the structure DIALOG+ offered. One participant noted:

You can adjust a little bit depending on the patient. Some don’t have the energy to go through everything, so they choose a few topics. You can be a bit flexible.

#### Benefits of structured conversations

Most practitioners described the structure within the DIALOG+ intervention as supportive and clarifying. It also seemed to balance the conversation, making it more purposeful and concrete for both patients and practitioners. Some practitioners perceived that using DIALOG+ might be useful in situations where patients struggled with motivation or concentration, where the structured format seemed to help them stay focused and engaged. They also described that the person-centred structure may ease the MHPs’ burden of having to lead the conversation entirely on their own, instead allowing the patient’s own priorities to steer the conversation:

It actually becomes easier for us when the patient gets to take the lead. Otherwise, you sit there trying to come up with improvement areas just to construct a goal. This way, you don’t have to. I feel like I can just follow along—and that’s quite a relief.

Some practitioners also noted that the structured and visual nature of DIALOG+ could be particularly helpful for patients who benefited from clarity and concrete feedback. As one participant illustrated:

So we’ve looked at this group of patients with comorbidities, those on the autism spectrum, and then it becomes really clear and concrete to work with the computer. And a high-functioning patient I work with just said, ‘This is the best thing I’ve done. I mean, this is bloody great.’ ‘It becomes so clear,’ he said, ‘when I sit and look at this. Look here now! I can compare, and we can go back,’ because he’s done three or four follow-ups from the first rating. And being able to follow the patient in different life domains—that’s what I think is good.

The intervention was also described as helping shift attention away from a purely diagnostic or problem-based view of the patient:

Often, we only see a psychiatric patient. But there’s a person there as well, with many different needs… And we haven’t always managed to do a good enough job helping them move forward in life. In that context, this (DIALOG+) can be a complement. A way to see other sides of the person.

## Discussion

The findings suggest that the structure within DIALOG+ appears to change how routine conversations in psychosis outpatient care are conducted. When patients are invited to rate different life domains, select priorities, and participate in defining actions, this appears to affect how agendas are set and how responsibility is negotiated between patients and MHPs. Therefore, DIALOG+ may enable more patient-led discussions, joint reflection on needs, and possible actions. At the same time, some practitioners raised concerns that increased patient influence prompted them to reconsider their professional responsibilities during the encounter. The present study complements our previous qualitative study exploring patients’ experiences of DIALOG+ by examining the perspectives of mental health practitioners, thereby providing insight into both sides of the clinical encounter.

### Redistribution of agenda-setting and professional authority

A central finding suggests that a shift in power often occurs when patients themselves set the agenda for the conversation. Some practitioners described stepping back from predefined clinical agendas and allowing patient-selected life domains to guide the discussion. This aligns with previous research showing that individuals with psychosis value meaningful involvement in decisions concerning their care [5, 11, 14].

Situations were more complex when patients’ ratings diverged from practitioners’ clinical assessments. These tensions are well-known barriers to shared decision-making in psychiatric care, where clinicians may question patients’ capacity to make the “right decisions” and the potential risks this may entail [9, 13]. Some practitioners expressed uncertainty about whether patient-led agenda-setting might steer conversations away from issues they considered clinically most important, prompting them to reflect on their professional roles and responsibilities, as well as on what should be prioritised in the encounter. These reflections illustrate the tension that often characterises shared decision-making in psychosis care.

Shared decision-making in psychosis has been described as positioned between paternalistic and fully autonomous models [23]. The findings suggest that DIALOG+ does not remove professional responsibility and appears to function best when MHPs’ expertise and patients’ perspectives are negotiated collaboratively. This may be facilitated by the therapeutic relationship itself. Ahmed et al., [1] identified the therapeutic relationship as a key enabler of shared decision-making and found that therapeutic relationships and trust enabled professionals to discuss risk with service users and collaborate with them in risk management. In this sense, the DIALOG+ intervention appears compatible with collaborative models that emphasise concordance between clinical expertise and patient-defined values (Treichler et al., 2021 [25]). This is consistent with Farrelly et al., [9], who argue that successful implementation of shared decision-making requires clinicians to develop communication processes that support collaborative dialogue rather than relying solely on traditional clinician-led consultations. Similarly, Kaminskiy et al., [13] describe shared decision-making as a process built on honest dialogue, valuing patients’ experiential knowledge, and recognising clinical encounters as a meeting of different forms of expertise rather than solely professional authority. Differences between patients’ and practitioners’ perspectives may serve as a starting point for dialogue. Making these differences explicit creates opportunities to explore the reasons behind them and to develop a shared understanding that can support collaborative decision-making. From this perspective, differing ratings are not necessarily indicators of disagreement, but may represent valuable clinical information that would otherwise remain unaddressed.

### Structure as enabler and constraint

The structured format was often experienced as both supportive and, in some cases, limiting. The structure appeared to focus conversations, clarify goals, and allocate responsibility for actions. This corresponds well with previous evaluations involving both patients and practitioners [16, 19, 20]. At the same time, some patients had significant cognitive difficulties and therefore lacked the capacity to understand or engage with the approach, which has also been demonstrated in earlier studies [19].

The results indicate that structure in itself does not guarantee involvement, but may depend on how the structure is translated and adapted in each individual encounter. For some practitioners, the structured format functioned as a framework for the clinical encounter rather than a rigid sequence that had to be completed in every session. Practitioners who described using the structure flexibly, adapting it to patients’ individual needs, preferences, and abilities while maintaining its overall purpose, generally described fewer challenges associated with the structured approach. Several practitioners described being surprised that DIALOG+ worked well with patients whom they had initially assumed would struggle to understand or manage the structured approach. There appears, therefore, to be a risk that clinicians’ assumptions about which patients the intervention is suitable for may function as a gatekeeping mechanism, limiting access to patients who might otherwise benefit from the approach.

Practitioners also described the visual presentation of ratings over time as an important aspect of the structured approach. Graphical comparisons of wellbeing between sessions made change visible and concrete for patients. In the context of psychotic disorders, which often involve changes in experiences and symptom severity over time [24], such longitudinal feedback may help to make change and progression visible. At the same time, it may strengthen the patient’s sense of agency and motivation. Through continuous follow-up and visual feedback, DIALOG+ may encourage patients to recognise their own progress, reinforcing participation in care over time. Through continuous follow-up and reflection in each session, DIALOG+ also appears to strengthen continuity and shared responsibility for agreed goals and actions. This aligns with the original design of DIALOG+ as a person-centred and solution-focused intervention [20, 21].

### Operationalising co-production in routine clinical encounters

A national evaluation in Sweden found that the implementation and use of person-centred methods are insufficient and vary across regions (Socialstyrelsen, 2022 [22]). Co-production has been described as collaboration in the design and delivery of healthcare, where patients and professionals jointly create value [2, 3]. At the individual level, this involves a collaborative co-production cycle, in which patients and clinicians jointly assess the situation, set priorities, and design care plans based on patient-defined goals [8]. The findings suggest that the use of DIALOG+ during routine encounters may provide a structured framework for such collaboration. By requiring patients to prioritise life domains and participate in defining actions, the intervention appears to support elements of the co-production cycle described by Elwyn et al., [8].

### Implications for professional roles and implementation

It appears important not to introduce DIALOG+ merely as a digital tool. It seems equally important to identify strategies for managing the power shift that often occurs, and that consequently leads to changes in the professional role. Previous studies have shown that there are barriers to person-centred approaches. These primarily concern time constraints, patients’ decision-making capacity, and the potential risks associated with patients making decisions [9, 13]. Co-production can be described not only as a procedural change but also as a relational and cultural transformation [2, 3]. Supporting practitioners in using the intervention flexibly according to the patient’s capacity, managing shifts in power, and balancing patient autonomy with practitioners’ own responsibilities may be important for sustainable implementation.

### Strengths and limitations

This study has several strengths. It explored practitioners’ experiences across five outpatient clinics within a specialised psychosis service and captures perspectives from multiple professional roles. The use of focus groups enabled participants to reflect together, which may have deepened the discussions compared to individual interviews. The reflexive thematic analysis provided an interpretative, pattern-oriented account of practitioners’ experiences, supported by iterative discussions within the research team and the use of NVivo to manage data. Credibility of the findings was strengthened by the alignment with a recent patient study from the same services [16].

Several limitations should also be considered. The sample size of 14 participants from a single department may limit transferability beyond Swedish specialist psychosis care. Although 31 practitioners were invited, little is known about reasons for non-participation beyond time constraints and travel issues. As participation was voluntary, self-selection bias cannot be ruled out. Focus group dynamics may also have shaped what was said, for example through alignment with group norms or dominance by more confident speakers. Methodologically, reflexive thematic analysis values researcher subjectivity as an analytic resource, but findings are nonetheless interpretative. Despite ongoing reflexivity, there is always a potential risk that the researchers’ professional backgrounds and familiarity with the setting may have influenced theme development. Finally, data were collected during an active implementation phase of DIALOG+, which may have intensified both enthusiasm and critical reflections. Different experiences may evolve as the intervention becomes more embedded in routine care.

## Conclusions

In summary, the findings suggest that DIALOG+ supports the operationalisation of co-production in outpatient psychosis care by giving patients greater influence over the agenda in routine clinical encounters. The intervention contributes to creating a structure in which differences between patients’ preferences and practitioners’ clinical assessments become visible and can be negotiated. Although the structured format supports patient involvement, its impact depends on how it is translated into clinical practice. Supporting practitioners in navigating shifts in professional roles and authority may therefore be important for realising the full potential of the intervention in psychosis care.

## Supplementary Information

Below is the link to the electronic supplementary material.

Supplementary Material 1

Supplementary Material 2

Supplementary Material 3

## Data Availability

To protect participant confidentiality (as specified in the informed consent), the data are not publicly available. Reasonable requests to the corresponding author will be considered, and a confidentiality assessment will be performed for each request.
