# Supplementary material for: Letting patients lead? Practitioners’ experiences of using DIALOG+ in psychosis outpatient care – patient involvement, collaborative dynamics, and professional roles
Source: BMC Psychiatry. 2026 Sep 17;26:766. doi: 10.1186/s12888-026-08633-6 (PMC13651122; doi:10.1186/s12888-026-08633-6)
Supplement: Supplementary file 1 [file 12888_2026_8633_MOESM1_ESM.docx]

**Consolidated criteria for reporting qualitative studies (COREQ): 32-item checklist**

| **No. Item** | **Guide questions/description** | **Comments &**  **Reported on Page #** |
| --- | --- | --- |
| **Domain 1: Research team and reﬂexivity** |  |  |
| *Personal Characteristics* |  |  |
| 1. Interviewer/facilitator | Which author/s conducted the interview or focus group? | ACA and MC moderated the focus group interviews (one conducted virtually). |
| 2. Credentials | What were the researcher’s credentials? E.g. PhD, MD | ML – MSc in Quality Improvement; ACA – PhD, senior researcher; AG – PhD, psychologist; MC – PhD, Psychologist. |
| 3. Occupation | What was their occupation at the time of the study? | ML – Doctoral student, Specialist psychiatric nurse, Business development manager at Sahlgrenska University Hospital; ACA – Senior researcher at Jonkoping Academy; AG – Psychologist at Sahlgrenska University Hospital; MC – Psychologist at Sahlgrenska University Hospital. |
| 4. Gender | Was the researcher male or female? | ML – male; ACA – female; AG – male; MC – female. |
| 5. Experience and training | What experience or training did the researcher have? | ML has extensive experience in psychosis care and qualitative research; ACA has expertise in qualitative research and improvement science; AG has research experience in Learning Health Systems and in the adaptation of DIALOG+; MC? |
| *Relationship with participants* |  |  |
| 6. Relationship established | Was a relationship established prior to study commencement? | ML had prior familiarity with some participants; ACA and MC had no prior relationship with participants. |
| 7. Participant knowledge of the interviewer | What did the participants know about the researcher? e.g. personal goals, reasons for doing the research | Participants were informed about the researchers’ professional roles and the purpose of the study during recruitment and before each focus group. |
| 8. Interviewer characteristics | What characteristics were reported about the interviewer/facilitator? e.g. Bias, assumptions, reasons and interests in the research topic | Reflexivity was emphasised throughout the study. Researchers discussed how their professional backgrounds and prior experiences could influence interpretation of the data. |

| **Domain 2: study design** |  |  |
| --- | --- | --- |
| *Theoretical framework* |  |  |
| 9. Methodological orientation and Theory | What methodological orientation was stated to underpin the study? e.g. grounded theory, discourse analysis, ethnography, phenomenology, content analysis | Reflexive Thematic Analysis (Braun & Clarke, 2022). |
| *Participant selection* |  |  |
| 10. Sampling | How were participants selected? e.g. purposive, convenience, consecutive, snowball | Purposive sampling – mental health practitioners who had conducted at least three DIALOG+ sessions. |
| 11. Method of approach | How were participants approached? e.g. face-to-face, telephone, mail, email | Participants were invited via their outpatient clinics. |
| 12. Sample size | How many participants were in the study? | 14 participants. |
| 13. Non-participation | How many people refused to participate or dropped out? Reasons? | A total of 31 practitioners were invited to participate. It is unclear why some chose not to take part, but several cited time or travel constraints. |
| *Setting* |  |  |
| 14. Setting of data collection | Where was the data collected? e.g. home, clinic, workplace | Department of Psychotic Disorders, Sahlgrenska University Hospital, Sweden; one focus group conducted virtually. |
| 15. Presence of non-participants | Was anyone else present besides the participants and researchers? | Only participants and moderators were present. |
| 16. Description of sample | What are the important characteristics of the sample? e.g. demographic data, date | 14 practitioners (7 nurses, 5 support workers, 2 social workers); 9 women, 5 men; experience range 1–44 years (mean 13.5 years). |
| *Data collection* |  |  |
| 17. Interview guide | Were questions, prompts, guides provided by the authors? Was it pilot tested? | A semi-structured interview guide developed by ML and refined with ACA, MC and AG; informed by prior patient interviews. |
| 18. Repeat interviews | Were repeated interviews carried out? If yes, how many? | None. |
| 19. Audio/visual recording | Did the research use audio or visual recording to collect the data? | All interviews were audio- or video-recorded. |
| 20. Field notes | Were ﬁeld notes made during and/or after the interview or focus group? | Field notes were taken during each focus group to capture contextual observations and researchers’ reflections on group dynamics and emerging impressions. |
| 21. Duration | What was the duration of the interviews or focus group? | 60–100 minutes (average 77 minutes). |
| 22. Data saturation | Was data saturation discussed? | In line with reflexive thematic analysis, the concept of data saturation was not applied. Instead, analysis continued until sufficient depth and diversity of meaning were achieved across the data set. |
| 23. Transcripts returned | Were transcripts returned to participants for comment and/or correction? | Transcripts were not returned to participants for comment or correction. |
| **Domain 3: analysis and ﬁndings** |  |  |
| *Data analysis* |  |  |
| 24. Number of data coders | How many data coders coded the data? | Two (ML and MC). |
| 25. Description of the coding tree | Did the authors provide a description of the coding tree? | A summary of themes and sub-themes (reflecting the coding structure) is presented in Table 1 under the Results section. |
| 26. Derivation of themes | Were themes identiﬁed in advance or derived from the data? | Themes were derived inductively from the data and refined through iterative discussions among researchers. |
| 27. Software | What software, if applicable, was used to manage the data? | NVivo 14 used for data management. |
| 28. Participant checking | Did participants provide feedback on the ﬁndings? | Participants did not provide feedback on the findings. |
| *Reporting* |  |  |
| 29. Quotations presented | Were participant quotations presented to illustrate the themes/ﬁndings? Was each quotation identiﬁed? e.g. participant number | Yes – quotations are presented to illustrate each theme and are anonymised. |
| 30. Data and ﬁndings consistent | Was there consistency between the data presented and the ﬁndings? | Yes – quotations and interpretations are consistent with the presented findings. |
| 31. Clarity of major themes | Were major themes clearly presented in the ﬁndings? | Yes – three major themes and six sub-themes are presented (see Table 1). |
| 32. Clarity of minor themes | Is there a description of diverse cases or discussion of minor themes? | Yes – variation and contrasting experiences are discussed, highlighting both enabling and constraining aspects of DIALOG+ use. |
