# Supplementary material for: Letting patients lead? Practitioners’ experiences of using DIALOG+ in psychosis outpatient care – patient involvement, collaborative dynamics, and professional roles
Source: BMC Psychiatry. 2026 Sep 17;26:766. doi: 10.1186/s12888-026-08633-6 (PMC13651122; doi:10.1186/s12888-026-08633-6)
Supplement: Supplementary file 2 [file 12888_2026_8633_MOESM2_ESM.docx]

**Description of the DIALOG+ intervention:**

DIALOG+ is a structured, computer-mediated intervention designed to support patient-centred communication, shared decision-making, and solution-focused care in community mental health settings. During a DIALOG+ session, patients are invited by clinicians to rate their satisfaction with eight life domains (e.g., mental health, physical health, job situation) and three treatment aspects (e.g., meetings with professionals and medication), highlighting areas where they need further support:

If DIALOG+ has been used before, current ratings can also be compared with previous ratings, providing a longitudinal perspective on the patient's progress. During the rating process, the patient also selects areas where further support is needed. Based on the overview of the current ratings, the patient and the clinician may identify and discuss the patient’s current concerns and which of these should be focused on during the meeting:

Concerns in each area are addressed in a 4-step solution-focused approach based on solution-focused therapy. The approach is intended to help patients and clinicians to understand the patient's concerns (‘understanding’), identify scenarios for improvement (‘looking forward’), explore options for actions (‘exploring’), and finally agree on actions for improving the patient's condition and social situation (‘agreeing’):

Finally, a summary of the DIALOG+ session is saved in the program. This summary, which includes the patient's self-assessed well-being and agreed-upon actions, can be printed and shared with the patient. At the next DIALOG+ session, previous ratings and agreed actions can be reviewed, supporting continuity, longitudinal monitoring, and ongoing collaborative care.
