# Supplementary material for: Letting patients lead? Practitioners’ experiences of using DIALOG+ in psychosis outpatient care – patient involvement, collaborative dynamics, and professional roles
Source: BMC Psychiatry. 2026 Sep 17;26:766. doi: 10.1186/s12888-026-08633-6 (PMC13651122; doi:10.1186/s12888-026-08633-6)
Supplement: Supplementary file 3 [file 12888_2026_8633_MOESM3_ESM.docx]

# Interview Guide – Focus Group Interview on Dialog+

## Informed Consent

The purpose of the interview is to understand clinicians’ experiences and perceptions of using Dialog+ with patients. The results will be published in a scientific article. This interview is part of a broader research project on Dialog+ that will contribute to a future doctoral thesis.

Participation in this study is entirely voluntary. You may choose not to participate, or to withdraw from the interview at any time without providing a reason. Your decision to participate or not participate will not affect you in any way.

The interview will be recorded and analysed. No identifying information will be collected or reported, and all quotations used in publications will be anonymised. Only members of the research group will have access to the recorded material. The recording will be deleted once the analysis is complete and the results have been published.

The information collected in this study will only be used for research purposes.

I have read the information above and consent to participate in this study and to the use of anonymised information and quotations in publications arising from this research:

Name: ________________________________________________

Date: _________________________________________________

## Interview Guide for Focus Group

This focus group discussion aims to explore the experiences and perceptions of practitioners regarding the DIALOG+ intervention.

- How many times have you used Dialog+?
- What is your overall experience of working with the intervention?
- What advantages and disadvantages do you see for yourself as a practitioner?
- What advantages and disadvantages do you see for the patient?

### Experiences of conducting the assessment with patients

The patient initially rates their well-being and their experience of care across 11 areas.

- Advantages, disadvantages, and challenges
- Does it contribute to patient involvement? If so, how?
- Are there any areas within Dialog+ that are particularly difficult or that you feel are missing?

### Experiences of discussing patient ratings

Your experience of discussing the patient’s ratings and addressing the areas the patient wants to focus on during the continued conversation.

- The patient chooses which areas to focus on. What are your thoughts on that? Advantages, disadvantages, and challenges?
- What kind of issues or concerns tend to become the focus of the conversations?
- How do you experience comparing current ratings with previous ratings? What are the benefits and challenges?
- How do you think Dialog+ differs from other conversations – advantages and disadvantages?

### Solution-focused methodology

The conversation methodology is a solution-focused method that includes four steps (Shared understanding, In the future and beyond, Explore options, and Agree on actions).

- What are your experiences and impressions of working with a solution-focused method? Does it help patients solve problems?
- What aspects are experienced as positive, and what aspects are experienced as difficult, problematic, or challenging?
- Do you perceive the method as suitable for all patients? Why or why not?

### Different ways of using Dialog+

There are three different ways to use Dialog+. What are your experiences, perceptions, and thoughts regarding these approaches?

1. Conducting only the assessment with the patient (without the conversation methodology) to track patients’ well-being over time
2. Using Dialog+ to develop a person-centred care plan
3. Using Dialog+ generally for structured conversations

### Concluding questions

- In what ways does Dialog+ stand out?
- What does Dialog+ add to your work and to the organisation as a whole?
- What influences whether you continue or discontinue using Dialog+?
- Are there any other thoughts or reflections that we have not discussed today?
